# Dexmedetomidine Pretreatment of Neuronal Cells Has Protective Effect Against Cell Death During Oxygen-glucose Deprivation/Reoxygenation, Based on IGF-1 Production

**DOI:** 10.14789/jmj.JMJ23-0037-OA

**Published:** 2024-09-11

**Authors:** YUI YAMANE, XIAOJIA LI, KEI HANAFUSA, HITOSHI NAKAYAMA, KOJI WATANABE, KAZUHISA IWABUCHI, MASAKAZU HAYASHIDA

**Affiliations:** 1 Department of Anesthesiology and Pain Medicine, Juntendo University Graduate School of Medicine, Tokyo, Japan; 1 Department of Anesthesiology and Pain Medicine, Juntendo University Graduate School of Medicine, Tokyo, Japan; 2 Department of Anesthesiology and Pain Medicine, Juntendo University Urayasu Hospital, Chiba, Japan; 2 Department of Anesthesiology and Pain Medicine, Juntendo University Urayasu Hospital, Chiba, Japan; 3 Institute for Environmental and Gender-Specific Medicine, Juntendo University Graduate School of Medicine, Chiba, Japan; 3 Institute for Environmental and Gender-Specific Medicine, Juntendo University Graduate School of Medicine, Chiba, Japan; 4 Infection Control Nursing, Juntendo University Graduate School of Health Care and Nursing, Chiba, Japan; 4 Infection Control Nursing, Juntendo University Graduate School of Health Care and Nursing, Chiba, Japan; 5 Department of Anesthesiology, Itabashi Chuo Medical Center, Tokyo, Japan; 5 Department of Anesthesiology, Itabashi Chuo Medical Center, Tokyo, Japan

**Keywords:** dexmedetomidine, insulin-like growth factor 1, ischemia, neuroprotection

## Abstract

**Objective:**

Insulin-like growth factor 1 (IGF-1) protects neuronal-cell damage by ischemia. Although neuronal cells have been reported to produce IGF-1, the molecular mechanisms remains obscure. Dexmedetomidine (DEX) protects neuronal cells from ischemic damage. We investigated the involvement of IGF-1 in the effect of DEX pretreatment on neuronal ischemic damage using an *in vitro* mouse hippocampal neuron model.

**Materials:**

We used Dexmedetomidine and cryopreserved passaged mouse hippocampal neuronal HT22. Other reagents in this study were analytical grade.

**Methods:**

Ischemia-reperfusion was modeled using the *in vitro* oxygen-glucose deprivation/reoxygenation (OGD/R). The effect of DEX was examined by incubating cells in DEX-containing medium for 1 hour prior to OGD/R. The cell damages were evaluated by lactate dehydrogenase (LDH) release. The amount of released IGF-1 were evaluated quantitatively by ELISA. The degree of Akt phosphorylation was evaluated by western blotting.

**Results:**

OGD/R loading promoted LDH release from neuronal cells, while DEX pretreatment suppressed the LDH release. IGF-1 release from them was primed by DEX pretreatment under OGD/R condition, but not under normal conditions. Akt was activated in DEX-pretreated cells following OGD/R loading. IGF-1 neutralizing antibody (*α*IGF-1) eliminated the above effects of DEX pretreatment. However, IGF-1 receptor expression in neuronal cells was not affected by DEX pretreatment prior to OGD/R loading.

**Conclusions:**

Our results demonstrate that neuronal cells primed with DEX under OGD/R conditions could release IGF-1 and potentially protect themselves via the IGF-1/Akt pathway. Consequently, it appears that neuronal cells activated by DEX have the capacity to self-protect from ischemic damage.

## Introduction

Neuronal damage is commonly linked with cognitive dysfunction, which significantly affects quality of life and daily activities. Currently, there are no definitive treatments for cognitive dysfunction, making it crucial to explore neuroprotective mechanisms that prevent its occurrence. Research indicates that in various neurodegenerative diseases associated with cognitive decline, the Akt-mediated pathway plays some roles in the neuroprotective effects driven by insulin-like growth factor 1 (IGF-1)^1-3)^. IGF-1 is a vital biological agent that maintains neuronal balance under normal conditions by binding to its receptors on neuronal cells^4)^. Typically, IGF-1 levels in the cerebral cortex are low in healthy adult brains^5)^, with production primarily occurring outside the brain and the protein being transported to neurons^6)^. In instances of brain injury, neurons themselves have been reported to produce IGF-1, which then acts as an endogenous protective agent^7-10)^. Although the relationship between ischemic brain injury, which is another significant cause of neuronal damage, and IGF-1 has been extensively studied using models such as the OGD/R model^11)^, the dynamics of IGF-1 production and Akt activation specifically within neuronal cells during ischemia/reperfusion remain unreported.

Dexmedetomidine (DEX), a selective *α*2 adrenergic receptor agonist, interacts with receptors in the locus ceruleus at the dorsolateral pons to reduce noradrenaline release and maintain gamma-aminobutyric acid (GABA) release in the hypothalamus, thus inhibiting histamine release^12)^. Given its minimal respiratory effects, DEX is frequently used to provide sedation, analgesia, and anxiolysis in elderly patients with multiple medical issues. Although temporary hypotension and cerebral hypoxia during general anesthesia can lead to postoperative delirium, which is a form of cognitive dysfunction^13, 14)^, DEX has been shown to decrease the incidence of postoperative cognitive dysfunction^15)^. Furthermore, DEX mitigates ischemia-induced neuronal injury^16)^. *In vivo* experiments have shown that neuronal cells produce IGF-1 under anesthesia with medetomidine, a stereoisomer of DEX^10)^.

These observations raise the possibility that using DEX may also protect neuronal cells from ischemia/reperfusion injury and mitigate postoperative cognitive dysfunction preemptively during general anesthesia. In this study, to elucidate the molecular mechanisms underlying DEX’s protective effects on neuronal injury in the context of oxygen-glucose deprivation/reperfusion (OGD/R), we explored the impact of DEX pretreatment on neuronal cell injury using an *in vitro* OGD/R model.

## Materials and Methods

### Materials

Dexmedetomidine (DEX; (+)-(S)-4-[1-(2,3- dimethylphenyl)ethyl]-1H-imidazole monohydrochloride), in the form of DEX hydrochloride, was from Cayman Chemical (Ann Arbor, MI, USA). cOmplete protease inhibitor cocktail was from Merck (Darmstadt, Germany). Mouse recombinant IGF-1 and goat anti-mouse IGF-1 neutralizing polyclonal IgG (*α*IGF-1) were from R&D Systems (Minneapolis, MN, USA). Rabbit anti-pAkt and anti-Akt were from Cell Signaling Technology (Danvers, MA, USA). Anti-mouse *β*-actin was from Santa Cruz Biotechnology (Dallas, TX, USA). Horseradish peroxidase (HRP)-conjugated goat anti-rabbit and anti-mouse IgG secondary antibodies were from Proteintech (Rosemont, IL, USA). Pre-analyzed gas mixtures were from Nippon Megacare Co. (Tokyo, Japan).

### Cell culture

Cryopreserved passaged mouse hippocampal neuronal HT22 cells were purchased from Merck Millipore (Tokyo, Japan) and maintained in high glucose DMEM supplemented with 10% fetal bovine serum (FBS), 1% penicillin/streptomycin, and 1% L-glutamine. HT22 cells were added to 6-well plates at a concentration of 1×106 cells/cm2 per well.

### Experimental treatments

*In vitro* oxygen-glucose deprivation/reoxygenation (OGD/R) method, which provides a useful model of glial scarring following *in vivo* general cerebral infarction and reperfusion (GCI/R)^17)^, was performed as described previously^17-19)^. In brief, HT22 cells cultured in high-glucose DMEM within 6-well plates were incubated with or without 5 nM DEX for 1 h, followed by washing with PBS. Subsequently the cells were cultured in glucose-free DMEM. Anoxia was induced by placing the cells in a modular incubator chamber (MIC-101; Billups- Rothenberg; Del Mar, CA, USA), flushing with pre-analyzed 95% N_2_ / 5% CO_2_ gas mixture for 15 min, and bubbling with the same gas mixture at flow rate 3 L/min. After incubating for 4 hours at 37°C in the anaerobic chamber, the cells were subsequently cultured in high-glucose DMEM for an additional 24 hours in a normal atmospheric condition. In some cases, cells were incubated following OGD in the presence or absence of 10 ng/ml IGF-1 recombinant protein and/or 500 ng/ml *α*IGF-1 as indicated.

In previous studies, plasma concentration of DEX that preserves cognitive function was estimated to range from 0.7-1.3 ng/ml^20)^, equivalent to concentration 2.9-5.5 nM in distribution volume 2-3 L/kg, and DEX concentration 1-100 μg/kg (2.5-250 nM in distribution volume 2-3 L/kg) was used to obtain neuronal protective effect during ischemia in animal models^21)^. Accordingly, we set DEX concentration in this study at 5 nM, a clinical dose that was expected to provide neuronal protective effects but was less than doses in most previous *in vitro* studies^22-25)^.

**Figure 1:**
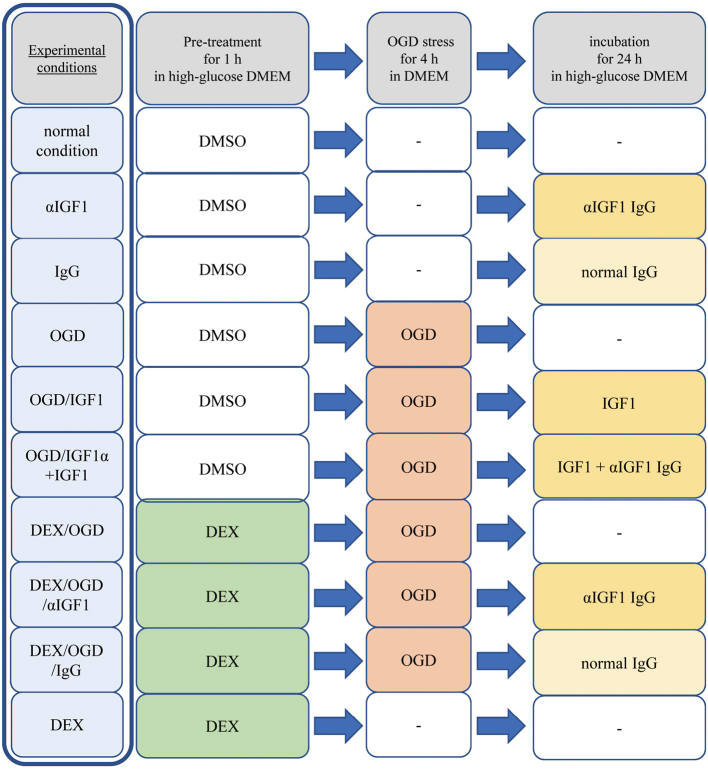
Scheme chart of the experimental conditions used in this study The experimental scheme chart illustrates the various conditions employed in the oxygen-glucose deprivation/reperfusion (OGD/R) experiment with HT22 cells. Initially, the cells were pretreated with either 5 nM Dexmedetomidine (DEX) or without any treatment for 1 hour in high-glucose Dulbecco's Modified Eagle Medium (DMEM). Following the DEX pretreatment, cells were either cultured in glucose-free DMEM under OGD conditions or maintained in high-glucose DMEM under a normal atmosphere for 4 hours. Subsequently, all groups of cells were further incubated for 24 hours in high-glucose DMEM under a normal atmosphere. During this phase, some cells were treated as indicated with either 10 ng/ml recombinant insulin-like growth factor 1 (IGF-1), 500 ng/ml anti-mouse IGF-1 neutralizing polyclonal IgG (αIGF-1 IgG), or normal IgG. The vehicle used was 0.005% (v/v) DMSO, with DEX dissolved in the same concentration of DMSO.

Experimental procedures and conditions are summarized in figure 1.

### Lactate dehydrogenase (LDH) release

Neuronal cell death (apoptosis) was quantified based on amount of LDH released from HT22 cells into medium, using cytotoxicity LDH assay kit- WST (Dojinsha; Tokyo, Japan) as per manufacturer’s instructions. Culture supernatants were collected, and LDH assays were performed using a microplate reader (Infinite M2000; TECAN; Männedorf, Switzerland).

### Enzyme-linked immunosorbent assay (ELISA)

IGF-1 concentrations in conditioned medium of HT22 were determined using DuoSet ELISA Development Systems (R&D Systems), with absorbances at 450 nm measured by microplate reader (model 2030 ARVOTM; PerkinElmer; Yokohama, Japan).

### Western blotting

HT22 cells were lysed and total protein extracted using lysate buffer (10 mM Tris pH 7.5, 150 mM NaCl, 5 mM EDTA, 5% (v/v) cOmplete, 10 mM NaF, 2 mM Na_3_VO_4_, 1 mM DFP). Protein concentrations were measured using BCA protein assay kits (Thermo Fisher; Waltham, MA, USA). Proteins were separated by 10% SDS-PAGE under reducing conditions and transferred to PVDF membranes. Membranes were blocked with Super Block (Thermo Fisher) for 1 h at room temperature, incubated overnight at 4°C with primary antibodies (rabbit anti-pAkt (1:2000), rabbit anti-Akt (1:2000), rabbit anti-IGF-1R (1:1000), mouse anti-*β*-actin (1: 10000)), washed, incubated with HRP-conjugated secondary antibodies for 1 h at room temperature, developed with SuperSignal West (Thermo Fisher), and exposed to X-ray film. For each protein, optical density was analyzed using Image J software program (National Institutes of Health; http://rsb.info.nih.gov/ij/), and normalized relative to *β*-actin. Degree of phosphorylation of Akt protein was calculated as ratio of band intensity of phosphorylated protein to that of protein.

### Statistical analysis

Data were expressed as mean ± SD from three or more independent experiments, and compared using ANOVA. Differences with p < 0.05 were considered statistically significant.

## Results

### DEX pretreatment protected HT22 cells from damage due to OGD/R

**Figure 2:**
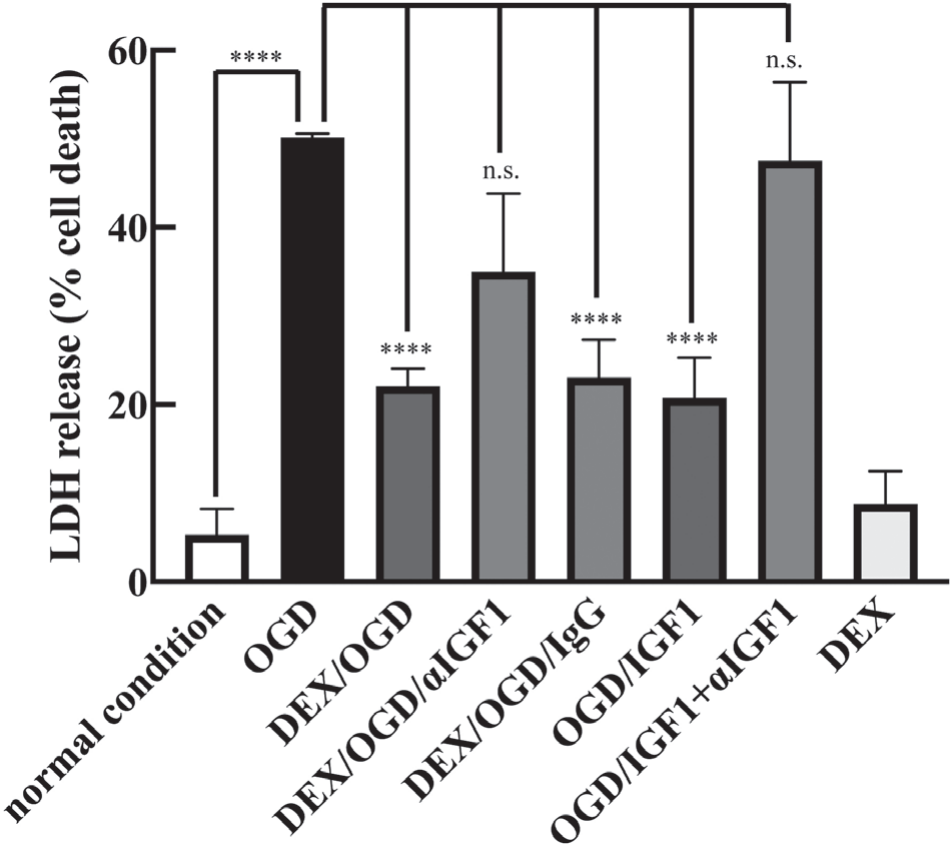
Effects of DEX on OGD/R-induced LDH release from HT22 cells Experimental conditions are outlined in Figure 1. After the incubation for 24 hours under normal atmospheric conditions, neuronal cell death was quantified by measuring the amount of lactate dehydrogenase (LDH) released from HT22 cells into the medium, as described in the Materials and Methods section. Data shown are mean ± SD from three independent experiments. *p < 0.05; ****p < 0.0001.

Direct protective effects of DEX pretreatment against neuronal damage were verified by measuring enzymatic activity of LDH in mouse hippocampal neuronal HT22 culture supernatants. These assays showed that oxygen-glucose deprivation/ reoxygenation (OGD/R) increased LDH release in the culture supernatants by 50.2 ± 0.4% (Figure 2). In contrast, OGD/R-induced LDH release was reduced 22.1 ± 1.9% by DEX pretreatment, and 20.8 ± 4.6% by IGF-1 treatment. Treatment with *α*IGF-1 during reoxygenation suppressed the inhibitory effect of DEX pretreatment on LDH release. Under these experimental conditions, neither DEX pretreatment (Figure 2) nor *α*IGF-1 (data not shown) affected cell viability.

### DEX pretreatment primed IGF-1 production

**Figure 3:**
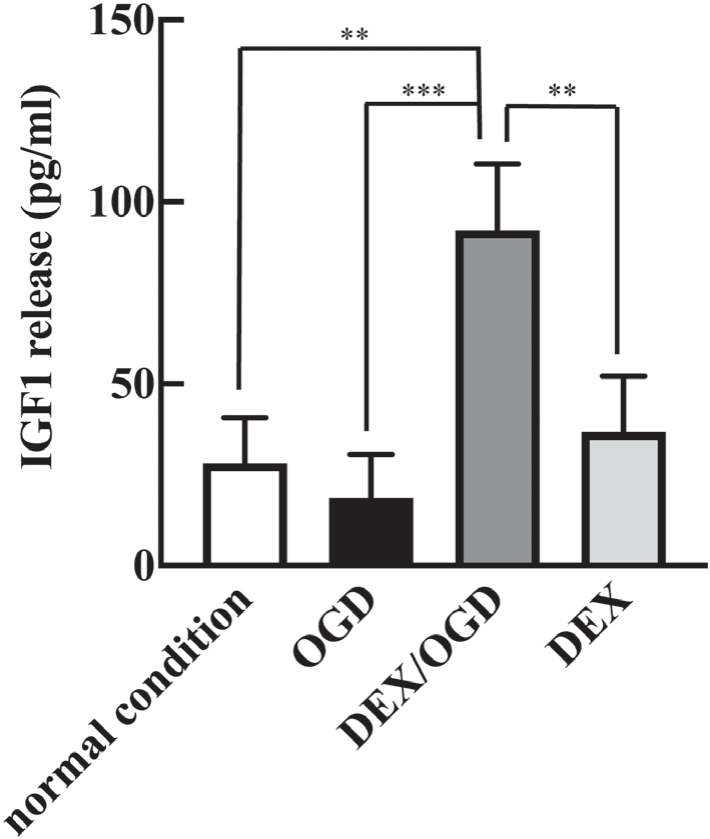
Effects of DEX on OGD/R-induced IGF-1 production by HT22 cells After 24-hour incubation under normal atmospheric conditions, cell supernatants were collected, and IGF-1 concentrations were determined using ELISA. The experimental conditions are described in Figure 1. Data shown are mean ± SD from three independent experiments. **p < 0.01; ***p < 0.001.

To elucidate whether DEX induces IGF-1 production from HT22 cells, IGF-1 contents in the culture supernatants were measured by ELISA. IGF-1 release from HT22 cells was not enhanced by OGD/R alone (Figure 3). The same result was true for the DEX-pretreated cells. However, IGF-1 was released extracellularly from DEX-pretreated and OGD/R-loaded HT22 cells.

### DEX pretreatment did not prime IGF-1R expression on HT22 cells

**Figure 4:**
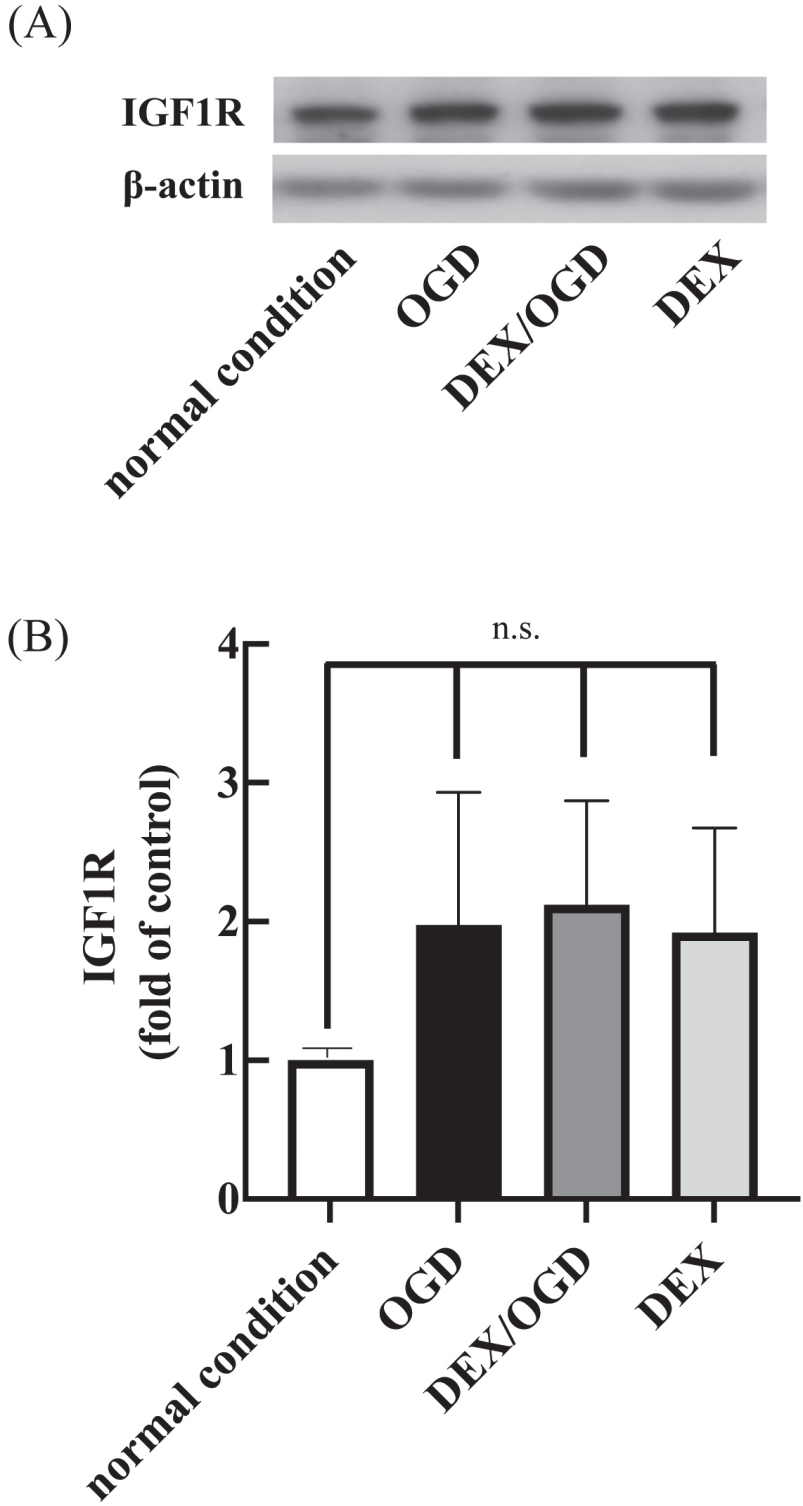
Effects of DEX on OGD/R-induced insulin-like growth factor 1 receptor (IGF-1R) production by HT22 cells (A and B) After 24-hour incubation under normal atmospheric conditions, cells were lysed, and analyzed by Western blotting sequentially with anti-IGF-1R and anti-β-actin antibodies (A). Experimental conditions are outlined in Figure 1. Blots are representative of three independent experiments. Relative band intensities were calculated based on densitometric analysis (B). Data shown are mean ± SD from three independent experiments. n.s., not significant.

To evaluate the effect of DEX pretreatment on IGF-1 receptor (IGF-1R) expression of HT22 cells during OGD/R, we analyzed the production of IGF-1R by western blotting. The results indicated a slight, but not statistically significant, upregulation in IGF-1R expression due to OGD/R loading and DEX pretreatment. Unlike IGF-1 release, there was no synergy in DEX-pretreatment and OGD/R-loading on IGF-1R expression (Figure 4).

### DEX pretreatment activated IGF-1/PI3K/Akt pathway

**Figure 5:**
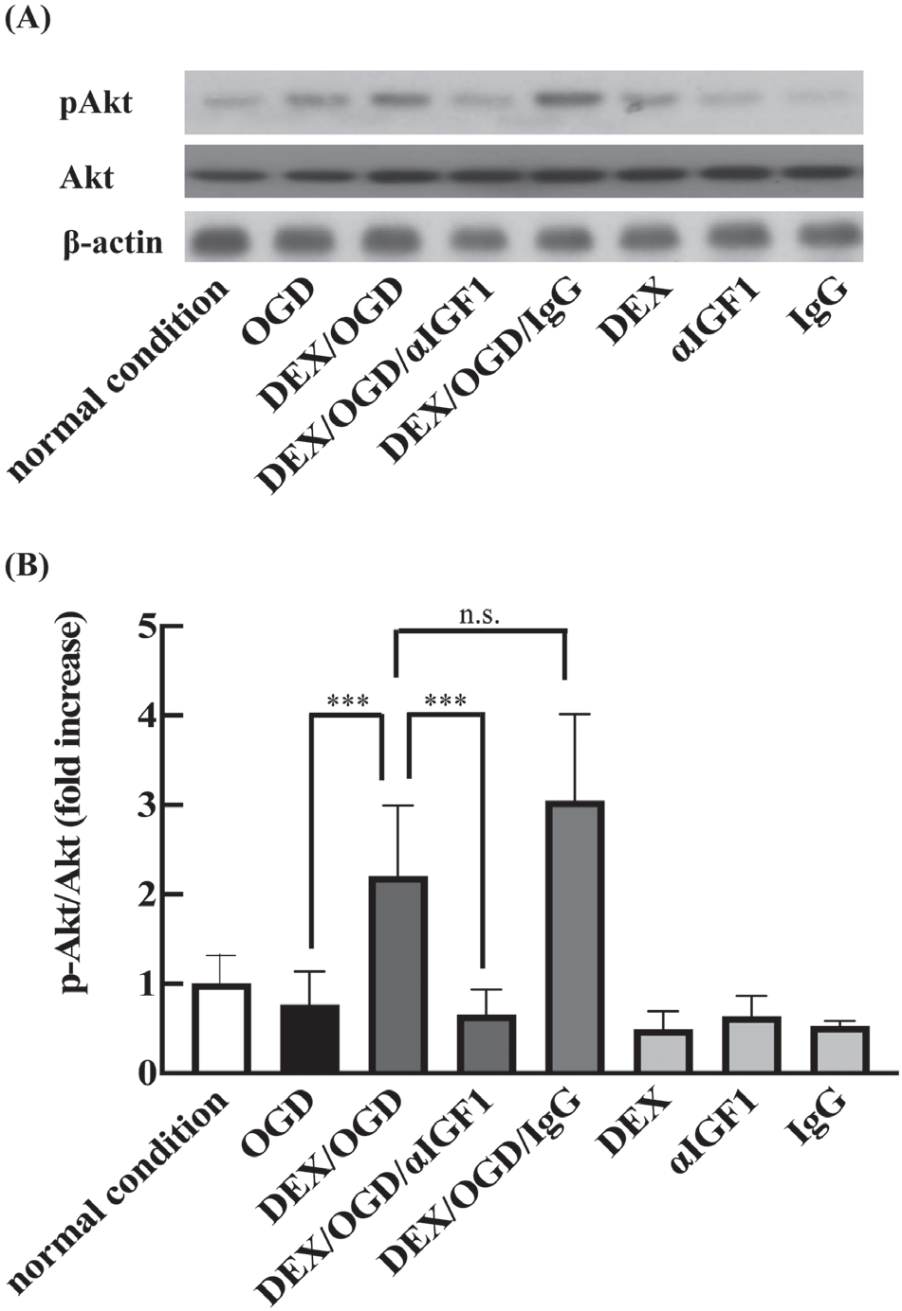
Effects of DEX on OGD/R-induced Akt pathway activation in HT22 cells (A and B) After 24-hour incubation under normal atmospheric conditions, cells were lysed and analyzed by Western blotting using antibodies against pAkt, Akt, and β-actin (A). The blots shown are representative of three independent experiments. The amount of Akt proteins was normalized to β-actin as an internal control. The ratios of phospho-Akt to Akt were calculated and compared with findings under normal conditions (B). Experimental conditions are outlined in Figure 1. Data are presented as mean ± SD from three independent experiments. ***p < 0.001; n.s., not significant.

The effect of DEX on Akt activation was evaluated by Western blotting. Akt activity after OGD/R for 24 h was not notably changed compared to control cells, but was significantly increased in DEX-pretreated cells (Figure 5). *α*IGF-1 treatment suppressed such DEX-induced Akt activity, to a level similar to that in OGD/R-loaded cells. Without OGD/R, DEX or *α*IGF-1 did not affected Akt activity.

## Discussion

Our findings as above demonstrate that DEX- primed HT22 cells produced IGF-1 under OGD/R condition but not normal conditions, and protected themselves against OGD/R-induced cell death.

We selected HT22, an immortalized mouse hippocampal cell line widely used for studies of neurodegenerative diseases and strokes^26, 27)^, as an *in vitro* model for assessing effects of OGD/R. Moreover, DEX pretreatment has been reported to protect against cell damage in the hippocampus, whereas DEX treatment after ischemia did not protect against neuronal damage *in vivo*^28)^. These cells released LDH extracellularly under OGD/R, and such LDH release was inhibited by DEX pretreatment. DEX has a preventive effect on brain damage caused by ischemia^16)^. Using a mouse model of intestinal ischemic, DEX treatment before induction of ischemia was found to result in a higher survival rate than DEX treatment after induction of ischemia^29)^. These results suggest that DEX is a useful agent for preventing against neuronal damage induced by ischemia. In our experiments, DEX was not present in culture medium during OGD/R. DEX pretreatment protects against neuronal cell death in hippocampus; however, DEX treatment following ischemia did not have such protective effect^28)^. It therefore appears likely that DEX pretreatment results in acquisition by neuronal cells of a protective effect against OGD/R. Typically, it has been demonstrated that ischemic injury induces an inflammatory reaction in tissues surrounding the cerebral infarction area, leading to further damage in healthy tissue^30)^. This suggests the possible critical importance of early medical intervention to counteract ischemic inflammation, with the aim of possibly minimizing disruption and potentially facilitating early recovery^31)^. In summary, while it might be that DEX has limited effectiveness in treating existing nerve damage, it appears to hold significant potential for preventing new injuries in healthy nerves near the affected area.

IGF-1 is both an internal and external factor in protection against neuronal cell death^7, 10, 32)^. The protective effect of DEX on OGD/R-induced LDH release from HT22 cells was suppressed by *α*IGF-1 treatment. Such LDH release was inhibited by IGF-1 treatment, similarly to DEX pretreatment. These findings indicate that DEX-pretreated cells release IGF-1 during OGD/R, even when DEX is absent from the medium. DEX-pretreated cells released IGF-1 protein into culture supernatant under OGD/R condition, but not under normal conditions. OGD/R loading *per se* did not induce IGF-1 release by cells. Thus, it seems that DEX pretreatment evidently promotes IGF-1 production by these cells under OGD/R conditions. Our findings indicate that IGF-1 protects neuronal cells from death under OGD/R conditions. This observation is consistent with *in vivo* studies that suggest neurons produce IGF-1 as a neuroprotective agent in damaged brains, functioning in an autocrine or paracrine manner^7-10)^. In our experiments, the anti- IGF-1 neutralizing antibody abolished the protective effect of DEX pretreatment on neuronal cell death under OGD/R conditions. Therefore, it appears that DEX-pretreated neuronal cells release IGF-1 and protect themselves via an IGF-1-dependent survival system under OGD/R conditions, even though the amount of IGF-1 produced by the neurons is negligible.

DEX has been reported to protect against ischemia-induced neuronal damage via *α*2 adrenergic receptors^33, 34)^. However, no study to date (to our knowledge) has addressed involvement of *α*2 adrenaline agonists in IGF-1 production. Molecular mechanisms whereby DEX pretreatment promotes IGF-1 production activity in neuronal cells under OGD/R condition remain to be elucidated. One possibility is that DEX “preconditions” cells to a state capable of IGF-1 production under ischemic stress, via *α*2 adrenergic receptors.

IGF-1/PI3K/Akt signaling system is generally considered to protect neuronal cells from ischemia- induced cell death. We therefore hypothesize that IGF-1/PI3K/Akt signaling system is responsible for DEX pretreatment-dependent neuroprotective effect under OGD/R. We observed Akt activation in DEX-pretreated cells under OGD/R condition but not under normal conditions, and such activation was suppressed by *α*IGF-1. OGD/R loading *per se* did not result in Akt phosphorylation in our model. We therefore hypothesize that IGF-1/PI3K/Akt signaling system is responsible for DEX pretreatment-dependent neuroprotective effect under OGD/R.

IGF-1 production in brain has been observed in several *in vivo* studies^7, 10)^. Elimination half-life of DEX is 2.1-3.1 h^35)^, suggesting that DEX may be a useful anesthetic agent for prevention of neuronal cell death induced by perioperative ischemia. Some anesthetics, including isoflurane and propofol, are known to promote rather than prevent neuronal cell death *in vivo*, but DEX was reported to counteract this effect via PI3K/Akt pathway^36, 37)^. Similarly, DEX protected against isoflurane- or bupivacaine-induced apoptosis of HT22 cells via PI3K/Akt pathway *in vitro*^25)^. Amelioration of neuronal cell death by IGF-1/PI3K/Akt signaling system has been reported in certain cognitive disorders (Parkinson’s disease, Huntington’s disease)^1-3)^. Mechanisms of post-operative cognitive impairment remain unclear, but are presumed to involve ischemia-induced neuronal cell death^38-40)^. Therefore, pretreatment with DEX as anesthetic has the potential to inhibit postoperative delirium (POD) induced by neuronal cell death caused by ischemia or other anesthetics during the perioperative period.

In conclusion, our findings demonstrate that *α*2_A_- adrenergic molecule-primed neuronal cells possess to release IGF1, activate Akt, and protect by themselves from damage under ischemia. However, how the *α*2A-adrenergic molecules cause neuronal cells to produce IGF1 needs to be studied in the future.

## Funding

This study was supported in part by Grant-in- Aid (S1311011) from MEXT Supported Program for the Strategic Research Foundation at Private Universities, and by institutional sources.

## Author contributions

YY conceptualized this study, methodized, investigated, formally analyzed, and was a major contributor in writing the manuscript. XL, KH and HN contributed in western blotting experiments. KW. methodized. KI mainly conceptualized this study, supervised, and wrote, reviewed and edited the manuscript. MH supervised, and reviewed and edited the manuscript. All authors read and approved the final manuscript.

## Conflicts of interest statement

The authors declare that there are no conflicts of interest.

## References

[1] Humbert S, Bryson EA, Cordelières FP, et al: The IGF-1/Akt pathway is neuroprotective in Huntington's disease and involves Huntingtin phosphorylation by Akt. Dev Cell, 2002; 2: 831-837. 12062094 10.1016/s1534-5807(02)00188-0

[2] Yang L, Wang H, Liu L, et al: The Role of Insulin/IGF-1/PI3K/Akt/GSK3beta Signaling in Parkinson's Disease Dementia. Front Neurosci, 2018; 12: 73. 29515352 10.3389/fnins.2018.00073 PMC5826217

[3] Fernandez AM, Jimenez S, Mecha M, et al: Regulation of the phosphatase calcineurin by insulin-like growth factor I unveils a key role of astrocytes in Alzheimer's pathology. Mol Psychiatry, 2012; 17: 705-718. 22005929 10.1038/mp.2011.128

[4] Dyer AH, Vahdatpour C, Sanfeliu A, et al: The role of Insulin-Like Growth Factor 1 (IGF-1) in brain development, maturation and neuroplasticity. Neuroscience, 2016; 325: 89-99. 27038749 10.1016/j.neuroscience.2016.03.056

[5] Bach MA, Shen-Orr Z, Lowe WL Jr, et al: Insulin-like growth factor I mRNA levels are developmentally regulated in specific regions of the rat brain. Brain Res Mol Brain Res, 1991; 10: 43-48. 1647481 10.1016/0169-328x(91)90054-2

[6] Nishijima T, Piriz J, Duflot S, et al: Neuronal activity drives localized blood-brain-barrier transport of serum insulin-like growth factor-I into the CNS. Neuron, 2010; 67: 834-846. 20826314 10.1016/j.neuron.2010.08.007

[7] Lee WH, Clemens, JA, et al: Insulin-like growth factors in the response to cerebral ischemia. Mol Cell Neurosci, 1992; 3: 36-43. 19912843 10.1016/1044-7431(92)90006-n

[8] Komoly S, Hudson LD, Webster HD, et al: Insulin-like growth factor I gene expression is induced in astrocytes during experimental demyelination. Proc Natl Acad Sci U S A, 1992; 89: 1894-1898. 1371885 10.1073/pnas.89.5.1894 PMC48560

[9] Costales J, Kolevzon A: The therapeutic potential of insulin-like growth factor-1 in central nervous system disorders. Neurosci Biobehav Rev, 2016; 63: 207-222. 26780584 10.1016/j.neubiorev.2016.01.001 PMC4790729

[10] Walter HJ, Berry M, Hill DJ, et al: Spatial and temporal changes in the insulin-like growth factor (IGF) axis indicate autocrine/paracrine actions of IGF-I within wounds of the rat brain. Endocrinology, 1997; 138: 3024-3034. 9202248 10.1210/endo.138.7.5284

[11] Li SL LJ, Zhou HS, Xiong LL: Research progress of IGF-1 and cerebral ischemia. Ibrain, 2021; 28: 57-67. 10.1002/j.2769-2795.2021.tb00066.x PMC10528794 37786870

[12] Nelson LE, Lu J, Guo T, Saper CB, Franks NP, Maze M: The alpha2-adrenoceptor agonist dexmedetomidine converges on an endogenous sleep-promoting pathway to exert its sedative effects. Anesthesiology, 2003; 98: 428-436. 12552203 10.1097/00000542-200302000-00024

[13] Wang X, Feng K, Liu H: Regional cerebral oxygen saturation and postoperative delirium in endovascular surgery: a prospective cohort study. Trials, 2019; 20: 504. 31412906 10.1186/s13063-019-3586-y PMC6694555

[14] Maheshwari K, Ahuja S, Khanna AK, et al: Association Between Perioperative Hypotension and Delirium in Postoperative Critically Ill Patients: A Retrospective Cohort Analysis. Anesth Analg, 2020; 130: 636-643. 31725024 10.1213/ANE.0000000000004517

[15] Yang W, Kong LS, Zhu XX, et al: Effect of dexmedetomidine on postoperative cognitive dysfunction and inflammation in patients after general anaesthesia: A PRISMA-compliant systematic review and meta-analysis. Medicine (Baltimore), 2019; 98: e15383. 31045788 10.1097/MD.0000000000015383 PMC6504304

[16] Hoffman WE, Kochs E, Werner C, et al: Dexmedetomidine improves neurologic outcome from incomplete ischemia in the rat. Reversal by the alpha 2-adrenergic antagonist atipamezole. Anesthesiology, 1991; 75: 328-332. 1677549 10.1097/00000542-199108000-00022

[17] Wang R, Zhang X, Zhang J, et al: Oxygen-glucose deprivation induced glial scar-like change in astrocytes. PLoS One, 2012; 7: e37574. 22629422 10.1371/journal.pone.0037574 PMC3358261

[18] Wang K, Zhu Y: Dexmedetomidine protects against oxygen-glucose deprivation/reoxygenation injury-induced apoptosis via the p38 MAPK/ERK signalling pathway. J Int Med Res, 2018; 46: 675-686. 29210287 10.1177/0300060517734460 PMC5971521

[19] Zhu C, Zhou Q, Luo, et al: Dexmedetomidine Protects Against Oxygen-Glucose Deprivation-Induced Injury Through Inducing Astrocytes Autophagy via TSC2/mTOR Pathway. Neuromolecular Med, 2020; 22: 210-217. 31654225 10.1007/s12017-019-08576-0 PMC7230061

[20] Ebert TJ, Hall J, Barney JA, et al: The effects of increasing plasma concentrations of dexmedetomidine in humans. Anesthesiology, 2000; 93: 382-394. 10910487 10.1097/00000542-200008000-00016

[21] Huang R, Chen Y, Yu AC, et al: Dexmedetomidine-induced stimulation of glutamine oxidation in astrocytes: a possible mechanism for its neuroprotective activity. J Cereb Blood Flow Metab, 2000; 20: 895-898. 10894172 10.1097/00004647-200006000-00001

[22] Liu YJ, Wang DY, Yang YJ, et al: Effects and mechanism of dexmedetomidine on neuronal cell injury induced by hypoxia-ischemia. BMC Anesthesiol, 217; 17: 117. 28854873 10.1186/s12871-017-0413-4 PMC5577810

[23] Sun YB, Zhao H, Mu DL, et al: Dexmedetomidine inhibits astrocyte pyroptosis and subsequently protects the brain in in vitro and in vivo models of sepsis. Cell Death Dis, 2019; 10: 167. 30778043 10.1038/s41419-019-1416-5 PMC6379430

[24] Zhang X, Wang J, Qian W, et al: Dexmedetomidine inhibits tumor necrosis factor-alpha and interleukin 6 in lipopolysaccharide-stimulated astrocytes by suppression of c-Jun N-terminal kinases. Inflammation, 2014; 37: 942-949. 24429914 10.1007/s10753-014-9814-4

[25] Bao F, Kang X, Xie Q, et al: HIF-*α*/PKM2 and PI3K-AKT pathways involved in the protection by dexmedetomidine against isoflurane or bupivacaine-induced apoptosis in hippocampal neuronal HT22 cells. Exp Ther Med, 2019; 17: 63-70. 30651766 10.3892/etm.2018.6956 PMC6307527

[26] Zhang Y, Zhang Y, Jin XF, et al: The Role of Astragaloside IV against Cerebral Ischemia/Reperfusion Injury: Suppression of Apoptosis via Promotion of P62-LC3-Autophagy. Molecules, 2019; 24: 1838. 31086091 10.3390/molecules24091838 PMC6539971

[27] Jung YS, Weon JB, Yang WS, et al: Neuroprotective effects of Magnoliae Flos extract in mouse hippocampal neuronal cells. Sci Rep, 2018; 8: 9693. 29946137 10.1038/s41598-018-28055-z PMC6018738

[28] Kuhmonen J, Pokorný J, Miettinen R, et al: Neuroprotective effects of dexmedetomidine in the gerbil hippocampus after transient global ischemia. Anesthesiology, 1997; 87: 371-377. 9286902 10.1097/00000542-199708000-00025

[29] Zhang XY, Liu ZM, Wen SH, et al: Dexmedetomidine administration before, but not after, ischemia attenuates intestinal injury induced by intestinal ischemia-reperfusion in rats. Anesthesiology, 2012; 116: 1035-1046. 22417965 10.1097/ALN.0b013e3182503964

[30] Shichita T, Ooboshi H, Yoshimura A: Neuroimmune mechanisms and therapies mediating post-ischaemic brain injury and repair. Nature Rev Neurosci, 2023; 24: 299-312. 36973481 10.1038/s41583-023-00690-0

[31] Ramos-Cabrer P, Campos F, Sobrino T, et al: Targeting the ischemic penumbra. Stroke, 2011; 42: S7-11. 21164112 10.1161/STROKEAHA.110.596684

[32] Gluckman P, Klempt N, Guan J, et al: A role for IGF-1 in the rescue of CNS neurons following hypoxic-ischemic injury. Biochem Biophys Res Commun, 1992; 182: 593-599. 1370886 10.1016/0006-291x(92)91774-k

[33] Ma D, Hossain M, Rajakumaraswamy N, et al: Dexmedetomidine produces its neuroprotective effect via the alpha 2A-adrenoceptor subtype. Eur J Pharmacol, 2004; 502: 87-97. 15464093 10.1016/j.ejphar.2004.08.044

[34] Maier C, Steinberg GK, Sun GH, et al: Neuroprotection by the alpha 2-adrenoreceptor agonist dexmedetomidine in a focal model of cerebral ischemia. Anesthesiology, 1993; 79: 306-312. 8102042 10.1097/00000542-199308000-00016

[35] Weerink MAS Struys M, Hannivoort LN, et al: Clinical Pharmacokinetics and Pharmacodynamics of Dexmedetomidine. Clin Pharmacokinet, 2017; 56: 893-913. 28105598 10.1007/s40262-017-0507-7 PMC5511603

[36] Li Y, Zeng M, Chen W, et al: Dexmedetomidine reduces isoflurane-induced neuroapoptosis partly by preserving PI3K/Akt pathway in the hippocampus of neonatal rats. PLoS One, 2014; 9: e93639. 24743508 10.1371/journal.pone.0093639 PMC3990549

[37] Xiao Y, Zhou L, Tu Y, et al: Dexmedetomidine attenuates the propofol-induced long-term neurotoxicity in the developing brain of rats by enhancing the PI3K/Akt signaling pathway. Neuropsychiatr Dis Treat, 2018; 14: 2191-2206. 30214209 10.2147/NDT.S169099 PMC6118247

[38] Olotu C: Postoperative neurocognitive disorders. Curr Opin Anaesthesiol, 2020; 33: 101-108. 31764008 10.1097/ACO.0000000000000812

[39] Bilotta F, Lauretta M, Borozdina A, et al: Postoperative delirium: risk factors, diagnosis and perioperative care. Minerva Anestesiol, 20131; 79: 1066-1076. 23511351

[40] Wang N, Ma J, Liu J, et al: Histamine H3 Receptor Antagonist Enhances Neurogenesis and Improves Chronic Cerebral Hypoperfusion-Induced Cognitive Impairments. Front Pharmacol, 2019; 10: 1583. 32038255 10.3389/fphar.2019.01583 PMC6985542

